# “It’s about survival, love and care”—parents’ experiences of living with a child with ARFID: a Swedish interview study

**DOI:** 10.1186/s40337-025-01479-5

**Published:** 2025-12-16

**Authors:** Katarzyna Brimo, Oscar Cardona Castro, Helena Holmäng, Lisa Dinkler, Tove Lundberg, Maria Råstam, Beatrice Nyström

**Affiliations:** 1 Gillberg Neuropsychiatry Centre, Institute of Neuroscience and Physiology, University of Gothenburg, Gothenburg, Sweden; 2 Department of Child and Adolescent Psychiatry, Skåne University Hospital, Lund, Sweden; 3 Department of Medical Epidemiology and Biostatistics, Karolinska Institute, Stockholm, Sweden; 4 Department of Environmental Medicine, Kochi Medical School, Kochi University, Kochi, Japan; 5 Department of Clinical Sciences, Lund University, Lund, Sweden; 6 Department of Psychology, Lund University, Lund, Sweden

**Keywords:** ARFID, Eating disorders, Feeding problems, Child healthcare, Parent experience, Qualitative research, Thematic analysis

## Abstract

**Background:**

A child with avoidant/restrictive food intake disorder (ARFID) has a diet so limited that it leads to medical, nutritional, and/or psychosocial problems. ARFID affects both children and their families, often causing parents to feel anxious and isolated. This study aimed to explore the experiences of Swedish parents raising a child with ARFID.

**Method:**

Data were generated through semi-structured interviews with six parents of children diagnosed with ARFID. All children were undergoing treatment for ARFID at a paediatric healthcare clinic. The interview material was transcribed and analysed using thematic analysis with the NVivo 14 software.

**Results:**

The analysis resulted in three themes: (1) “It’s hard work”: ARFID dominating life, (2) “It’s not about forcing”: Seeking balance, and (3) “You feel questioned”: Feeling different. All themes highlighted the complex and demanding reality that parents faced. A significant part of the families’ daily lives revolved around managing the eating disorder, with parents facing numerous challenges related to their child’s eating difficulties. Parents attempted to strike a balance between planning and improvisation to manage mealtime stress and unpredictability. They struggled to understand their child’s eating habits, facing ongoing tension between promoting variety and accepting restrictions, as well as between voluntary eating and using pressure. Constant worry about the child’s health and development was compounded by a lack of understanding from others and difficulty accessing appropriate care and support.

**Conclusions:**

There is a need to raise awareness of ARFID and its related difficulties in early childhood. Detailed clinical guidelines for healthcare providers should be developed and implemented to enhance patient care. Parental experiences can serve as a valuable knowledge base for improving early detection and intervention strategies for families with ARFID (e.g., meal planning, addressing nutrition concerns with supplements, supporting parental responses).

**Plain English summary:**

This study looked at what it is like for Swedish parents to raise a child with avoidant/restrictive food intake disorder (ARFID), a condition where a child's extremely limited eating leads to health, nutrition, and/or social problems. Six parents were interviewed while their children were receiving treatment. Parents described life with ARFID as exhausting and all-consuming. Much of their daily routine revolved around planning meals, handling unpredictable eating behaviours, and worrying about their child's health. They tried to find a workable balance between planning meals and adapting on the fly, between encouraging new foods and accepting limitations, and between letting the child eat voluntarily and feeling pressure to intervene. Many parents also felt misunderstood or judged by others, which added to their stress and sense of isolation. They often struggled to get the right support from healthcare services. The study concludes that ARFID needs to be better recognised early in childhood, and that clearer clinical guidelines are needed. Parents' experiences can help improve early identification and support for families, including advice on meal planning, nutrition, and how to respond to a child's eating challenges.

## Introduction

Eating and drinking are essential to life, and many social interactions revolve around food, including family meals, school lunches, parties, travel, and dining out with friends. Parenting a child who eats selectively or insufficiently is a complex and multifaceted challenge, with potentially profound effects on family life.

Avoidant/Restrictive Food Intake Disorder (ARFID) is a relatively new eating disorder diagnosis, first introduced in the Diagnostic and Statistical Manual of Mental Disorders, 5th ed. (DSM-5) in 2013 [1] and later included in the International Statistical Classification of Diseases, 11th ed. (ICD-11) [47]. It is characterized by restrictive or avoidant eating behaviours that lead to weight loss or, in children, inadequate weight gain, significant nutritional deficiency (sometimes requiring supplements), and/or psychosocial impairment.

Unlike other eating disorders, ARFID is not driven by concerns about body image [18]. Instead, food avoidance is typically rooted in one or more specific factors: heightened sensory sensitivity (e.g., to texture, smell, or taste), a lack of interest in food (e.g., due to low appetite), or fear of adverse physical consequences (e.g., choking or vomiting) [33, 40]. While ARFID can occur at any age, onset is most common in childhood [1]. Most individuals with ARFID are underweight due to reduced calorie intake; however, some may have a normal or even high body weight if their restricted diet includes energy-dense foods [2, 35, 42]. Regardless of weight, ARFID can lead to significant nutritional imbalances that necessitate medical and dietary support. It is important to distinguish ARFID from common fussy eating in typically developing children, as ARFID is more severe, persistent, and impairing [38].

Children with ARFID often have co-occurring medical issues such as constipation, reflux, nausea, or abdominal pain [14, 35, 48], and the condition frequently coexists with psychiatric or neurodevelopmental disorders [11, 31, 45].

Research suggests ARFID typically begins earlier than other eating disorders, like anorexia nervosa, and is more common in boys [8, 30, 37]. Its prevalence in the general population is estimated at 1–2% (Dinkler et al. 2023), [11, 42], with much higher rates (up to 64%) reported in clinical settings [35].

Several treatment models – often based on cognitive-behavioural or family therapy – have been proposed, including individual therapy, family-based approaches, and parental support interventions [12, 22, 36, 41]. When treating children with ARFID, the role of parents is central to the success of these interventions. Nevertheless, despite their critical involvement, parents often receive limited support and encounter unhelpful advice [3, 20].

[34] argue, effective care requires integrating clinical expertise with parents’ lived experiences, an approach that can be challenging in practice. Current research emphasises nutritional and growth support as the primary goals in treating young people with ARFID [16, 39]. However, healthcare providers continue to face difficulties in identifying effective, tailored support strategies. A significant lack of research on parents’ experiences hinders the development of informed, family-centred care.

Addressing this gap, the present study explores the lived experiences of parents raising a child with ARFID, with the aim of highlighting the everyday and systematic challenges they face.

## Methods

This study was conducted as part of the project *“Children who do not eat. Avoidant/restrictive food intake disorder (ARFID) in a clinical context”.* The Swedish Ethical Review Authority (DNR 2021-05280) approved the study.

### Epistemological positioning

The study employed a reflexive thematic analysis [4] within a critical realist framework. From this perspective, there is no objective reality that can be fully described – instead, it is always mediated by interpretation. Accordingly, we assumed that an ARFID diagnosis represents one aspect that shapes the lived realities of children and their parents. The knowledge generated through this study reflects how parents experience and interpret these challenges, while acknowledging that individual interpretations may differ. In line with critical realism, the research findings are viewed as shaped by the researchers’ interpretive judgments [32].

### Participants

All participants were parents of children referred to a child and adolescent medicine clinic in southern Sweden due to avoidant and/or restrictive eating behaviours. Written invitations were sent to ten families, and nine were contacted by phone to confirm interest and provide additional information. Seven parents agreed to participate, and ultimately, six were available for interview—a number consistent with recommendations for small-scale qualitative research (Braun and Clarke 2013).

All participants were biological parents (five mothers and one father), and the term “parent” is used throughout. The mean age of parents was 38, and the mean age of children (three girls and three boys) was 4.2 years. Children’s mean BMI z-score was − 1.23 (see Table 1 for group characteristics). All children showed very little interest in food and followed a highly restricted diet. Four children failed to achieve expected weight gain; two of them had nutritional deficiencies and depended on supplements and/or nutritional drinks. Five out of six children reported sensory issues related to the texture, appearance, or smell of food. Two out of six reported a lack of hunger sensation, and one child had orofacial problems (chewing and swallowing). All children were diagnosed with ARFID based on DSM-5 criteria. Each child had at least one additional diagnosis, but none of their medical conditions could better explain their eating difficulties. None of the children had identified neurodevelopmental conditions at the time of data collection. All families received support from a multidisciplinary team for children with eating issues, including a paediatrician, a paediatric nurse, a speech therapist, a dietitian, a psychologist, a physiotherapist, and a medical social worker. Interventions were tailored to the child’s specific difficulties. Typically, the dietitian provided nutritional plans, supplements, and follow-up appointments, while the speech therapist offered sensory and orofacial interventions. Specific guidance varied depending on the child’s age and the nature of their eating challenges.

Consistent with critical realism, the depth of data analysis was prioritized over sample size [28]. Given the study’s purpose and the purposive sample, further recruitment was deemed unnecessary. Real names of the children were replaced with pseudonyms.

**Table 1:** Characteristics of the group (*n* = 6) at the time of data collection

Categories	Mean (± SD; Range)
Weight (SDS) Height (SDS)	−0.87 (± 1.99; −3.0-2.5) −0.45 (± 1.95; −2.1-3.2)
BMI (SDS)	−1.23 (± 1.12; −2.6-0.1)

BMI: Body Mass Index; SDS: Standard deviation score (z-score) calculated according to WHO reference data for age and sex; SD: Standard deviation

### Procedures

Data were collected in September 2022 through semi-structured interviews, designed to capture rich, detailed accounts that may be missed in quantitative research [4]. The interview guide encouraged open-ended discussion of parents’ experiences [44].

To ease into potentially complex topics, interviews began with positive, descriptive questions about the child. Initial questions addressed the current eating situation and guided participants in reflecting on their lives with ARFID. Follow-up questions explored specific challenges and lessons learned (see Supplement for interview guide).

Parents chose the interview format: one took place at home, one at the clinic, three via a digital platform, and one by phone. All interviews were conducted by a member of the research team (OCC), who was completing a supervised clinical psychology program. Interviews lasted between 39 and 74 min (M = 59, SD = 11), and all were audio-recorded and transcribed verbatim.

### Data analysis

Analysis followed the six-step process for reflexive thematic analysis. One researcher conducted the initial analysis, starting with familiarisation and coding (Steps 1–2) using NVivo 14 [25]. Initial themes were generated (Step 3), then reviewed, refined, and named collaboratively by the first author and co-authors (Steps 4–5). This involved an in-depth discussion of themes, subthemes, and naming. The final sixth step involved writing up the results, which were reviewed and approved by all authors [5].

### Ethical issues

Written informed consent was obtained from all participants. Debriefing was conducted after each interview. If distress arose, the interviewer facilitated contact with either the supervising psychologist or Child Health Services for parental support.

## Results

Thematic analysis generated three main themes and seven subthemes (Table 2), illustrating the profound impact of ARFID on parents’ daily lives. The first main theme, *“It’s hard work”: ARFID dominating life*, captures the daily practical and emotional challenges of parenting a child with ARFID. The second main theme, *“It’s not about forcing”: Seeking balance*, reflects parents’ efforts to balance nutritional needs, mealtime strategies, and the parent-child relationship. The third main theme, *“You feel questioned”: Feeling different*, describes the sense of isolation and misunderstanding that parents experienced, particularly in healthcare settings and social interactions.

**Table 2:** Overview of themes and sub-themes

Themes	Sub-themes
“It’s hard work”: ARFID dominating life	• Planning and improvising to deal with stress • Struggling to understand the child’s eating habits and manage frustration • Constant concern for the child’s physical and social development
“It’s not about forcing”: Seeking balance	• Ideal variety versus pragmatic restrictiveness • Voluntary eating versus using feeding strategies
“You feel questioned”: Feeling different	• Experiencing prejudice and misunderstanding • Advocating for the child in healthcare

### “It’s hard work”: ARFID dominating life

This theme captures how ARFID pervades daily life, requiring constant planning, emotional regulation, and concern for the child’s health and development. Many participants described how they tried to plan and improvise to manage the stress ARFID created. They also illustrated how their children’s eating habits could be hard to understand and predict, sometimes making them feel frustrated, as described in the second subtheme. Finally, as described in the last subtheme, parents had many concerns about their children’s physical and social well-being.

#### Planning and improvising to deal with stress

Parents described meal planning as mentally exhausting and logistically challenging. Decisions about when and what to serve required attention to mood, food availability, and past eating patterns. One parent described the constant considerations that need to be taken:

What does she like to eat? Is it the right day? Is it the right time? When was the last time she had a little pot of yoghurt? […] because if she has a small yoghurt, she won’t eat for like two hours thereafter. So, there’s a lot of planning around the meal situation. (Alexandra, mother of a 2-year-old girl)

Tools like food diaries were used to plan meals and avoid setbacks, such as vomiting or car sickness:

A food diary is great because we write down the times of the day. Then we know we can’t drive off anywhere in the next few hours, because he’ll throw up in the car […] So, it’s timing, what you can eat, what you can drink, can we go away somewhere, can we not go, and then as I said before in case of illness or infection you can go back and check. (Helen, mother of a 4-year-old boy)

Even with preparation, many parents described having to improvise when their child’s cues appeared briefly or unpredictably. One of the parents noted the stress of capturing rare eating opportunities: “If I’m lucky, she shows some signs of hunger. Then I have to cook in panic to catch the moment”. During the mealtime itself, this parent also had to be creative to get the child to eat:

She wants to leave the table as soon as the vegetables are gone. Then, she tugs at her bib, ready to get up. That’s when I must step in and say something like, “No, but we’re not going to read a book or do a puzzle right now - we can draw a little instead.” From there, I’ll say, “Let’s draw an animal, and then you take a bite.” She follows along – we draw, and she takes a bite. Then I’ll say, “Okay, now find the next bite”, and bit by bit, she eats. It works, but only under demanding conditions. It also takes a lot from us. (Elvira, mother of a 2.5-year-old girl)

The considerable effort invested in mealtimes and the prolonged duration of eating activities had a negative impact on other aspects of daily life. Alexandra, mother of a 2-year-old girl, described that from all of this, “You get tired. You get tired”. After working “all day,” parents felt an obligation to “force” themselves to make eating fun (Elvira, mother of a 2.5-year-old girl) and had less free time to recharge. As a result, they experienced lower energy levels and a decline in overall well-being.

#### Struggling to understand the child’s eating habits and manage frustration

Despite careful preparation, children often refused food, leaving parents confused and discouraged. The variation in the intensity of ARFID symptoms across different meals, weeks, or even more extended periods led to unpredictability. At times, the child might eat a substantial amount, while at other moments, they would consume very little. Jessica, mother of a 4.5-year-old boy, gave an example of her child having “a sandwich for breakfast and then not wanting anything for the rest of the day. Moreover, that could go on for a whole week”. These scenarios perplexed some parents about how eating could be so easy during one meal and so difficult during the next. Some children rejected meals due to minor sensory factors, such as a hard pancake edge, adding to the unpredictability:

To anyone else, they were just pancakes. But for her, the edges on the pancake were a bit too hard […], in this case, she refuses to eat. […]. I cut the pancake in the middle so that the edges disappear. Then, she can’t eat it because the pancake does not look like it should. (John, father of a 5.5-year-old girl)

When verbal communication was limited, parents relied on expressions or gestures to interpret their children’s food preferences, often preparing multiple dishes to accommodate their changing needs. Parents worked hard to experiment with various strategies to encourage their child to eat, including offering multiple foods, altering preparation methods, adjusting serving times, using different dining environments, or using varying plates and cutlery: “You try everything, fun colours, shapes but it doesn’t help. There is no influence on her at all” (John, father of a 5.5-year-old girl). This relentless effort, often met with refusal, led to frustration, fatigue, guilt, and emotional exhaustion.

#### Constant concern for the child’s physical and social development

Parents expressed deep anxiety about their children’s growth, development, and social experiences. They often researched nutrition extensively and tailored meals to maximize intake. One parent expressed concern that the child might not be getting enough nutrition, worrying that “her brain is developing a lot, and is she getting everything?”. This led the parent to try to learn all about nutrition:

You try to learn and read a lot about nutrition and see if she is getting everything since she eats so little. Can we try tofu? Can we do this? Can we cook the food? I’ve become a kind of food expert so that we can give her the best. So, it’s hard work, I must say. (Elvira, mother of a 2.5-year-old girl)

Illnesses worsened ARFID symptoms, sometimes triggering vomiting and regression. One parent described how having a cold led her child to feel “the mucus here [points to throat], and then he doesn’t want to eat certain foods. Then he starts to gag and vomit”. The parent continued to explain:

He vomits and vomits and vomits, and then we must start from scratch because he doesn’t just vomit up medications. He vomits up the whole meal, and then he has an empty stomach again. A thin little boy who must then get nutritional drinks again, so it has been very hard. (Helen, mother of a 4-year-old boy)

Parents also worried about their child’s isolation during social events involving food, as well as the long-term effects on friendships and inclusion. “Visiting people’s homes is tough. We need to bring food or explain everything. It limits how much we socialize” (Eva, mother of a 7-year-old boy).

Over time, the worries for their child’s development aggravated parents’ anxiety, pressure, and stress. Many expressed concerns about whether these feeding issues would improve over time. They also worried about the future, especially about their child starting school, managing social situations, and maintaining adequate nutrition and energy for learning and play:

What happens then? I mean, in a year, she will start school. That’s also a bit of a concern because once school begins, you really do need a certificate for special diets, for example. And that’s a worry… Will she be running around all day at school without eating a single thing? (John, father of a 5.5-year-old girl).

For many parents, these worries extended beyond practical matters, touching deeply on hopes for their child’s independence, inclusion, and overall quality of life.

### “It’s not about forcing”: seeking balance

This theme reflects the daily dilemmas parents faced, how to encourage eating without creating negative associations or damaging the parent-child relationship. The balancing act entailed choosing between promoting dietary variety or accommodating their child’s food restrictions, as outlined in the first subtheme, and between respecting the child’s autonomy or adopting a more directive approach, as explored in the second subtheme.

#### Ideal variety versus pragmatic restrictiveness

Parents wanted to introduce variety for nutritional balance but often deferred to the child’s preferences to avoid rejection. During more challenging periods, nutritional supplements or preferred foods, even if limited, were prioritized to prevent weight loss and fatigue.

This pragmatic approach could mean having crisps for dinner or limiting oneself to only one type of food. Decisions of being pragmatic were also grounded in the challenges that arose when the children did not have enough energy, which this participant illustrated well: “You must always think through the situation to make sure she eats. A hungry child becomes tired and impatient” (John, father of a 5.5-year-old girl).

To address nutritional gaps during these difficult periods, health professionals recommended compensatory strategies such as food substitutes, nutritional drinks, or supplements. These measures provided parents with reassurance that their child would not go hungry or suffer from nutrient deficiencies due to ARFID. In addition to offering peace of mind, such interventions also helped stabilise the situation - prescription dietary drinks, for instance, played a key role in managing deficiencies and reducing immediate health risks.

Dietitians and psychologists also played a vital role in offering emotional support to parents by creating a safe space where they could share their struggles and receive reassurance that practical deviations from standard dietary guidelines were both understandable and acceptable. One parent described the support they received:

I talked to a psychologist at the hospital. You feel bad as a parent who only gives him the food he wants, but she [the psychologist] said it was still better that he got something than nothing at all. (Eva, mother of a 7-year-old boy)

#### Voluntary eating versus using feeding strategies

Most parents believed pressure made things worse but admitted to using feeding strategies such as coercion: “You don’t force her, but sometimes we say: “No ice cream unless you try it!” Usually, she’d rather go hungry” (John, father of a 5.5-year-old girl).

Parents also used distraction as a feeding strategy. While using distractions was often helpful, it could also lead to undesirable outcomes, such as preventing children from developing mindful eating habits. One parent described:

I usually feed her when she isn’t aware of it because she is into something else. She closes her mouth, gags, or shakes her head when she realizes she has been fed. And she blatantly refuses. I can’t get her to eat. Eventually, she starts crying and wants to leave the table. (Alexandra, mother of a 2-year-old girl)

Another strategy was to directly reward the child, even when this went against the parent’s own principles. This could mean allowing a child to eat in front of a TV or a tablet. One participant described their strategy as a temporary solution:

If I feel that nothing works, then he gets it [the iPad], just because he must get solid food and practice eating more. So, I still see it as a small gain that he eats well, then. Then, he can have his iPad for a little while. (Helen, mother of a 4-year-old boy)

Parents also questioned the fine line between encouragement and nagging, especially when it led to conflict or distress. Others found success by involving their children in decisions about food and allowing them to control small choices, such as selecting cutlery or dipping sauce, which helped reduce anxiety and increase motivation.

### “You feel questioned”: feeling different

This theme highlights the social and institutional challenges parents encountered, including stigma, misunderstanding, and difficulty accessing support. They perceived others as reacting negatively to their child’s eating habits, as described in the first subtheme. Although several parents had positive experiences with healthcare interactions, many also described challenges in obtaining proper support, as discussed in the next subtheme.

#### Experiencing prejudice and misunderstanding

Many parents described being judged by others, who assumed their child’s behaviour stemmed from poor parenting or lack of discipline. Elvira, mother of a 2.5-year-old girl, expressed: “People think we spoil her or that she’s not raised properly. It’s frustrating and hurtful”.

Mealtimes with extended family or friends often became emotionally charged, prompting some to avoid social settings altogether or hide accommodations like screen time during meals:

I want nothing more than to sit at the table and have a cozy family dinner, but my child eats more if he eats in front of the TV. I would feel ashamed to tell them this since I know they would question whether children should eat in front of the screen. (Jessica, mother of a 4.5-year-old boy)

These judgments led to shame and self-doubt. Even institutions like preschools sometimes failed to understand ARFID, questioning the child’s independence or the parents’ methods:

The preschool might say: “Oh, so she does not eat by herself?” And we’re like: “Yeah, we must feed her, otherwise she won’t get what she needs.” Family and friends might say, “What? Isn’t she going to eat? Is she that spoiled? Doesn’t she like what we’ve made?” […]. They almost act like we’ve spoiled her by only making certain foods or by letting her leave the table, like she’s poorly raised. That’s mostly how people seem to interpret this whole [ARFID] thing. (Elvira, mother of a 2.5-year-old girl)

A lack of understanding from preschool and school staff often led to children being excluded from the social environment during mealtimes. In some cases, children went hungry at preschool, prompting parents to compensate with food at home. “He unfortunately tends to stay hungry most of the day, then drinks gruel from a baby bottle right after preschool. He’s very hungry when he gets home, so it’s a problem” (Helen, mother of a 4-year-old boy).

#### Advocating for the child in healthcare

Parents described varied experiences with healthcare. Some professionals were supportive and proactive, offering personalised guidance and reassurance. Others seemed dismissive or relied too heavily on growth curves, overlooking the parents’ daily struggles.

Positive experiences were usually characterised by a collaborative approach to addressing their children’s difficulties and a feeling that the provider cared for the child’s overall well-being. Helen, mother of a 4-year-old boy, described how their nurse from child health services called regularly, and asked: “How is he doing? Then I talk a bit more generally about him, too, not just about the diet. So, it feels good”.

It was also important for parents to feel heard and validated in their concerns. One participant described how they felt about the nurse from child health services who responded supportively and provided hope:

Then I called her [the nurse] and said, “I want to come in and weigh her because I feel that it has been a tough month.” She took me in immediately. When the weight curve showed no progress, the nurse said, “Let’s start again, and we’ll see.” She gave me some tips, and we looked at recipes together, deciding to try scrambled eggs. (Alexandra, mother of a 2-year-old girl)

As Alexandra described in the quote above, feeling heard and understood helped parents trust their child’s healthcare providers. This sense of trust and support reassured them when eating became problematic.

However, not all interactions with healthcare were described as positive. Expressions such as “being on the warpath” and “going on the offensive” were used in the interviews to illustrate the challenges parents faced in having their concerns about the child’s eating difficulties acknowledged. According to the parents, a significant part of their advocacy has been conveying to health professionals that there was a problem with their child’s eating. They described situations where they felt unheard and unsuccessful in convincing professionals, which forced them to exert some form of pressure or seek alternative access to specialized care or a proper assessment of the child’s condition. Some wished that healthcare providers, childcare services, and schools had been more considerate of their child’s unique and challenging circumstances: “You already have a battle at home. You don’t need another one in healthcare”. (Elvira, mother of a 2.5-year-old girl)

Consequently, many parents felt unheard, and their concerns, observations, and unique situations were dismissed. Alexandra (mother of a 2-year-old girl) said: “I cried after every visit. I felt bad because nobody listened to what I said. You think you know your child best anyway, right?”. Overall, the effort to secure appropriate care and support placed an additional burden on parents.

## Discussion

This study explored the lived experiences of Swedish parents raising a child with ARFID, focusing on daily life and seeking support. Parents frequently described feeling isolated, anxious, and misunderstood. Despite numerous interventions, their child’s feeding difficulties often persisted, and ARFID gradually came to dominate family life. Mealtimes became a source of ongoing stress, with parents feeling relentless pressure to ensure their child meets basic nutritional needs. Repeated failed attempts to feed their child contributed to feelings of frustration and anxiety. The added burden of preparing special meals further depleted parents’ time and energy. These findings are consistent with previous research on paediatric feeding challenges [15, 23, 43].

The distinction between voluntary eating and using external eating strategies can be viewed through the lens of intrinsic versus extrinsic motivation, based on Self-Determination Theory (SDT) as explained by [6] in their framework for a responsive approach to child feeding. From an SDT perspective, voluntary eating corresponds with intrinsic motivation – children’s internal desire to eat driven by hunger, curiosity, and enjoyment. Conversely, coercive or instrumental feeding tactics reflect extrinsic control, which can undermine autonomy and self-regulation. Findings from this study show that parents’ main goal was always to help and support their children. However, they admitted they sometimes used feeding strategies that could increase mealtime stress (e.g., coercion, rewarding, distraction). Consistent with previous research in subclinical “fussy eating” populations [17, 19, 46], these strategies may have been driven by concern, guilt, or competing parental goals, such as making sure children get enough nutrition or keeping family harmony.

By systematising parents’ lived experiences, this study also provides a valuable resource for healthcare professionals. As reflected in the identified themes, families often sought medical support under considerable emotional strain, with urgent needs and high expectations. Positive healthcare experiences were characterized by collaboration, validation, and acknowledgment – elements that may be especially critical for parents who feel emotionally vulnerable when engaging with professionals. These findings align with earlier research that highlights parents’ difficulties in navigating healthcare services [21, 23, 26, 29].

Parents often described feeling exhausted and overwhelmed when seeking help, and many carried a sense of guilt or shame over their perceived failure to help their child. These emotions can act as barriers to effective communication with professionals. This, in turn, highlights the importance of early detection tools to identify ARFID before these difficulties escalate. Promising tools for early screening have already been developed and tested in Japan and Sweden [9, 10].

A complementary approach to mitigating these challenges could involve recognising and validating parents’ emotions. [24] emphasises that validating guilt and shame can empower individuals. For healthcare providers, this means actively listening to parents and recognising the legitimacy of their experiences. Validation can help de-escalate negative emotions and support more productive conversations and planning.

Beyond the clinical setting, parents reported a lack of social understanding and support, consistent with findings from studies on eating disorders [13, 15], subclinical eating difficulties [7], and paediatric feeding disorders [21]. In the case of ARFID, this lack of support may be particularly harmful, contributing to emotional isolation and compounding family stress. These findings underscore the need for a comprehensive system of support that acknowledges and validates the lived experiences of affected families. Misunderstandings and preconceived notions often arise in school and preschool environments, where collaboration is essential for a child’s well-being. When staff lack awareness, children may be excluded during mealtimes, sometimes going without food altogether. This often forces parents to compensate at home, a task made more difficult by the complexities of ARFID. As a result, parents not only face the challenges of managing the disorder but also carry the additional burden of justifying their choices and shielding their children from judgment.

While professional validation is essential, peer support can also play a meaningful role. Parents of children with mental health conditions, including eating disorders, express a strong need for peer support groups - yet this need remains largely unmet within current healthcare models [27]. For parents of children with ARFID, support groups could offer a safe space to share experiences, reduce isolation, and learn from others. Prior research shows that such groups can foster empowerment, understanding, and relief [15].

## Strengths and limitations

This study aimed to offer a methodologically robust and conceptually thoughtful contribution to the growing body of research on ARFID. By adopting a critical realist epistemology and applying Braun and Clarke’s well-established flexible approach to thematic analysis, we provide a transparent and reflective interpretation of the data.

The major strength of this research study is that it replicates many findings from studies on sub-clinical feeding challenges. It appears that some of the psychological and relational processes reported in subclinical avoidant eating may be more intense in ARFID to the extent that they require clinical intervention or professional support. The study also demonstrates strong clinical relevance. By centring parents’ lived experiences, it sheds light on the day-to-day challenges of supporting a child with ARFID – an area often overlooked in clinical practice. The insights gained can meaningfully inform the development of more responsive and family-centred care strategies.

However, several limitations should be acknowledged. Although the study aimed to provide transferable insights, caution is warranted when applying the findings to other contexts. First, all participants were referred to a medical clinic, which may have influenced the presentation of ARFID observed. Second, the sample consisted primarily of mothers, resulting in the underrepresentation of fathers’ experiences. Nonetheless, as mothers often serve as primary caregivers, their perspectives remain particularly valuable. Third, the children varied in age, which may have shaped how they and their parents experienced ARFID. At the same time, this diversity allowed for a more comprehensive view of how ARFID manifests across early childhood. Furthermore, given the heterogeneous nature of ARFID, shared themes were identified, alongside notable individual differences. For example, some parents emphasized dietary variety, whereas others prioritized adequate energy intake through supplements or preferred foods. Finally, cultural and systemic differences, such as healthcare structures and parenting norms, may limit the transferability of findings to other countries or contexts. These factors should be carefully considered when interpreting or applying the results beyond Sweden.

## Conclusion

ARFID is often mistaken for a phase of extreme picky eating or attributed to poor parenting. These misconceptions can leave parents feeling ashamed and guilty, especially when compounded by a healthcare system that may lack adequate knowledge or resources for supporting children with ARFID. Parents in this study described tireless efforts to advocate for their children while facing conflicting advice, minimal support, and widespread misunderstanding – experiences that were deeply exhausting and emotionally draining.

Addressing these challenges requires improved training and greater awareness of ARFID among primary care providers. Early identification is crucial and depends on the availability of clear clinical guidelines and educational tools to enable timely and effective intervention. Equally important is the inclusion of parents’ lived experiences in shaping detection and intervention strategies, ensuring that care systems are responsive to the real-world needs of affected families.

Intervention and policy efforts should prioritize supporting parents to manage the emotional and relational dimensions of feeding, particularly their concern, guilt, and anxiety about their child’s eating. Providing psychoeducation and responsive feeding guidance within ARFID-focused interventions can help parents move away from externally controlling strategies (e.g., pressure, persuasion, or distraction) toward approaches that nurture intrinsic motivation for eating and promote a calmer, more trusting feeding relationship.

Practical support is also key. This includes helping parents understand why their child’s eating may fluctuate from day to day (for example, due to changes in sensory or emotional regulation, appetite, or autonomy needs), addressing nutritional concerns through supplements when appropriate, and offering reassurance about flexible, inclusive meal planning.

Ensuring access to multidisciplinary teams with expertise in ARFID is also critical for delivering comprehensive, coordinated care. Future research should focus on developing and evaluating evidence-based interventions tailored to the unique needs of patients with ARFID and their families. Such efforts are vital for enhancing treatment outcomes, supporting family well-being, and reducing the broader burden associated with this disorder.

## Data Availability

Data will be made available by the authors upon reasonable request.
